# THE 4MS AGE-FRIENDLY FRAMEWORK PROVES SUCCESSFUL TO ENHANCING A CERTIFIED NURSING ASSISTANT TRAINING PROGRAM

**DOI:** 10.1093/geroni/igad104.3624

**Published:** 2023-12-21

**Authors:** Valerie Bearden, Robin McAtee

**Affiliations:** Oaklawn Center on Aging, Hot Springs, Arkansas, United States; University of Arkansas for Medical Sciences, Little Rock, Arkansas, United States

## Abstract

The Arkansas Geriatric Education Collaborative, a HRSA-funded Geriatric Workforce Enhancement Program grantee, partnered with a local Certified Nursing Assistant training program to develop a curriculum utilizing the 4Ms Framework of Age-Friendly Care of older adults. The curriculum was designed to empower CNA students and other direct care staff with knowledge of how to deliver more effective, person-centered care. This was accomplished by synthesizing current content with each of the 4Ms: What Matters, Medication, Mentation, and Mobility. During the introduction to person-centered care, strategies infused included instruction about how to have meaningful conversations about What Matters to the older adult. For Mobility, instruction was provided regarding safety and the use of adaptive equipment. Mentation was addressed by teaching the students ways to prevent and identify conditions such as depression and delirium. To include the “M” of medication, the students were taught about using non-pharmacological strategies for conditions such as insomnia and pain. The way all elements are intertwined and can interfere with What Matters was emphasized and examples provided. Of the 34 CNA students trained, 100% of students achieved both objectives during a clinical experience. Improvement of care was identified by a student as recognizing how to calm a frightened resident by using validation therapy. Another student reported the strategy of providing a bed bath in lieu of a shower to a resident who was unsteady because of a pain medication. Program development was completed in April, 2023, at which time it was put into practice and evaluated.

